# Physicochemical properties and gel-forming ability changes of duck myofibrillar protein induced by hydroxyl radical oxidizing systems

**DOI:** 10.3389/fnut.2022.1029116

**Published:** 2022-11-16

**Authors:** Xueshen Zhu, Xiandong Shi, Shaohua Liu, Ying Gu, Junya Liu, Qingquan Fu, Renlei Wang

**Affiliations:** ^1^Key Laboratory of Biological Functional Molecules of Jiangsu Province, College of Life Science and Chemistry, Jiangsu Second Normal University, Nanjing, China; ^2^School of Life Science, Nanjing Normal University, Nanjing, China; ^3^School of Food Science, Nanjing Xiaozhuang University, Nanjing, China

**Keywords:** duck myofibrillar proteins, hydroxyl radical, protein oxidation, gel-forming capacity, physicochemical changes

## Abstract

This paper focuses on the changes of physicochemical properties and gel-forming ability of duck myofibrillar proteins (DMPs) induced using hydroxyl radical oxidizing systems. DMPs were firstly extracted and then oxidized at various H_2_O_2_ concentrations (0, 4, 8, and 12 mmol/L) using Fenton reagent (Fe^3+^-Vc-H_2_O_2_) to generate hydroxyl radicals, and the effects of hydroxyl radical oxidation on the physicochemical changes and heat-induced gel-forming capacity of DMPs were analyzed. We observed obvious increases in the carbonyl content (*p* < 0.05) and surface hydrophobicity of DMPs with increasing of H_2_O_2_ concentrations (0–12 mmol/L). In contrast, the free thiol content (*p* < 0.05) and water retention ability of DMPs decreased with increasing H_2_O_2_ concentrations (0–12 mmol/L). These physicochemical changes suggested that high concentrations of hydroxyl radicals significantly altered the biochemical structure of DMPs, which was not conducive to the formation of a gel mesh structure. Furthermore, the gel properties were reduced based on the significant decrease in the water holding capacity (*p* < 0.05) and increased transformation of immobilized water of the heat-induced gel to free water (*p* < 0.05). With the increase of H_2_O_2_ concentrations, secondary structure of proteins analysis results indicated α-helix content decreased significantly (*p* < 0.05), however, random coil content increased (*p* < 0.05). And more cross-linked myosin heavy chains were detected at higher H_2_O_2_ concentrations groups through immunoblot analysis (*p* < 0.05). Therefore, as H_2_O_2_ concentrations increased, the gel mesh structure became loose and porous, and the storage modulus and loss modulus values also decreased during heating. These results demonstrated that excessive oxidation led to explicit cross-linking of DMPs, which negatively affected the gel-forming ability of DMPs. Hence, when processing duck meat products, the oxidation level of meat gel products should be controlled, or suitable antioxidants should be added.

## Introduction

Myofibrillar proteins are the most abundant and important proteins in duck breast tissue, accounting for 50–60% of the total protein. A key functional property of these proteins is their ability to form heat-induced gels, which provide good texture and mouthfeel to meat products (1). Therefore, factors affecting these physicochemical properties, such as protein oxidation, must be identified. Reactive oxygen species, the main initiators of protein oxidation, can be generated during the manufacture of meat products (2). Protein oxidation includes all reactions that result in the removal of electrons from protein-binding sites, including free individual amino acids and peptides are oxidized (3). Protein oxidation may lead to modification of protein side chain groups, cross-linking and aggregation of protein macromolecules, altering the physicochemical properties of proteins to affect the color, texture, and taste of meat and meat products. In the past two decades, the biochemical and functional properties of myofibrillar proteins have been widely examined *in vitro* (4, 5). At increasing hydroxyl radical concentrations, the carbonyl value and surface hydrophobicity of myofibrillar proteins typically exhibit increasing trends, and the free thiol content in myofibrillar proteins shows a decreasing trend, indicating an increased degree of protein oxidation. Gel hardness, chewiness, and elastic modulus are significantly negatively correlated with the concentration of hydroxyl radicals (6). Recently, Bao et al. (7) reported that oxidation of porcine myofibrils leads to the loss of free sulfhydryl groups and histidine residues and the formation of myofibrillar protein carbonyl groups, eventually leading to the formation of large cross-linked fractions. However, other studies reported contrasting results, particularly regarding the effect of oxidation on gel properties. Previous studies indicated that within a certain range, disulfide cross-linking promoted stiffness to some extent because aggregation and denaturation of myofibrillar components may affect the properties of myofibrillar protein gels (8). Shen et al. (9) investigated the effect of oxidation on myofibrillar protein gel properties and their ability to bind specific species. Their results demonstrated that with increasing H_2_O_2_ concentrations, DMPs tended to expose their internal hydrophobic amino acids, leading to enhanced aggregation and increased surface hydrophobicity. Li et al. (10) found that changes in the microstructure of myofibrillar protein gels caused by hydroxyl radical-induced oxidation altered the ability to form gels. Furthermore, Wang et al. (11) reported that hydrophobic interactions between protein molecules were significantly enhanced by oxidation, which was not conducive to formation of a gel network structure. Thus, protein oxidation does not always lead to a decrease in protein gel-forming properties and corresponding functional properties, and moderate protein oxidation may improve or enhance the functional properties of muscle proteins.

Duck meat and its products are widely consumed in southern China because of their high nustritional value, good taste, and low price. Numerous world-famous duck meat products have emerged, including roasts and salted ducks. However, the gelation properties of duck breast muscle remain unclear. Gelling properties depend on several intrinsic factors, including the meat species, muscle type, and fiber type (12). Researchers have used a hydroxyl radical system to simulate systematic muscle oxidation *in vitro* to better understand the mechanism underlying the effect of protein oxidation on meat gelation. The effects of hydroxyl radicals on the physicochemical susceptibility and gel properties of DMPs have not been evaluated in detail, in which further investigation is still needed. In this study, hydroxyl radicals were used to oxidize DMPs *in vitro*, the physicochemical properties and gel properties changes would been investigated aiming to provide new information for understanding the mechanism of protein oxidation in complex DMPs gel system.

## Materials and methods

### Sample and duck myofibrillar proteins preparation

Five duck carcasses were purchased from a local market in Lishui district, Nanjing, China. All chemicals were analytical grade. DMPs were extracted as described by Park et al. (13), with appropriate modifications. The pre-prepared duck breast was removed, thawed on ice for 30 min, and transferred to a 500 mL centrifuge bottle. The breast was homogenized in 5 × phosphate buffer I (pH 7.0, 100 mmol/L NaCl, 2 mmol/L MgCl_2_, 1 mmol/L EGTA, 10 mmol/L K_2_HPO_4_) for 3 min using a high-speed homogenizer (Ultra Turrax, IKA, Germany) and centrifuged at 4°C, 5,000 × *g* for 10 min; Supernatant was then discarded. The above steps were repeated three times. During this process, the sample was filtered through a double layer of gauze to remove excess connective tissue. Subsequently, homogenization was performed in 5 × phosphate buffer II (125 mmol/L NaCl, 2.5 mmol/L MgCl_2_, 1.25 mmol/L EGTA, 12.5 mmol/L K_2_HPO_4_ containing 1% Triton X-100), the mixture was centrifuged at 4°C, 5,000 × *g* for 10 min, and the resulting supernatant was discarded to remove membrane proteins. This process was repeated three times. Finally, washing was performed with 4 × 0.1 mol/L NaCl, followed by centrifugation at 4°C, 5,000 × *g* for 10 min, This process was repeated three times to obtain pure DMPs precipitates. The proteins were placed in a small beaker, sealed, and stored at 4°C until analysis.

### Hydroxyl radical-generation system and physicochemical properties of proteins

#### Oxidation of duck myofibrillar proteins

The protein precipitate was dissolved in phosphate buffer (0.6 mol/L NaCl, 10 mmol/L K_2_HPO_4_, pH 7.0). The protein concentration was determined using the Biuret method and was adjusted to 33 mg/mL. Protein solutions were then treated with Fenton's reagent [containing of 0.01 mmol/L ferric trichloride (FeCl_3_), 0.1 mmol/L ascorbic acid (V_C_), and different concentrations of hydrogen peroxide (H_2_O_2_; 0, 4, 8, and 12 mmol/L)]. After oxidation for 24 h at 4°C, 40 μL 500 mmol/L EDTA was added to terminate the reaction. Phosphate buffer (30 mL; 0.1 mol/L NaCl containing 10 mmol/L K_2_HPO_4_, pH 7.0) was added to wash precipitated proteins, followed by centrifugation at 4°C, 10,000 × *g* for 10 min. This process was repeated twice to obtain the final oxidized protein precipitate.

#### Determination of carbonyl and free thiol contents

The carbonyl content was determined as described by Soglia et al. (14), with appropriate modifications. Briefly, the protein precipitate obtained *via* oxidation termination was dissolved in phosphate buffer (0.6 mol/L NaCl containing 10 mmol/L K_2_HPO_4_, pH 7.0). The absorbance of the group treated with 2,4-dinitrophenylhydrazine was measured at 280 and 370 nm. The carbonyl content was calculated using 22,000 L/(mol·cm) as the molar extinction coefficient for conversion and expressed in units of nmol/mg protein, as shown in Equation (1). The average value was obtained by repeating three times for each treated samples.


(1) $$\begin{array}{l} \text{Carbonyl content}=\frac{\left[A_{370}-A_{370(blank)}\right]}{22000\times \left\{A_{280}-\left[A_{370}-A_{370(blank)}\right]\times 0.43\right\}}\;\\ \;\times 10^{6}[nmol/mg\;protein] \end{array}$$

The free thiol group content was determined using Ellman's 5,5′-dithiobis-(2-nitrobenzoic acid) colorimetric method according to the method described by Bao et al. (15). Briefly, the protein precipitate was dissolved by adding 0.6 mol/L NaCl containing 10 mmol/L K_2_HPO_4_ (pH 7.0), and the protein concentration was adjusted to 5 mg/mL. 5,5′-Dithiobis-(2-nitrobenzoic acid) reacted with the thiol group and was used for subsequent absorbance detection at 412 nm. The thiol content was calculated using 14,150 L/(mol·cm) as the molar extinction coefficient for conversion and expressed in units of nmol/mg protein, as shown in Equation (2) with 3 repetitions for each treated group.


(2) $$\begin{array}{l} \text{Free thiol group content}=\frac{\left[A_{412}-A_{412(blank)}\right]}{14150\times A_{280}}\;\\ \;\times 10^{6}[nmol/mg\;protein] \end{array}$$

#### Surface hydrophobicity measurement

Surface hydrophobicity was determined as described by Chelh et al. (16), with some modifications. The oxidized protein precipitate was dissolved in phosphate buffer (0.6 mol/L NaCl containing 10 mmol/L K_2_HPO_4_, pH 7.0), and the protein concentration was adjusted to 5 mg/mL. Briefly, 200 μL of bromophenol blue was added to 1 mL of protein solution, and absorbance was measured at 595 nm. The surface hydrophobicity was calculated using Equation (3) with 3 repetitions for each treated group.


(3) $$\begin{array}{l} \text{Surface hydrophobicity}=\frac{\left[{\text{A}}_{595\left(\text{blank}\right)}-{\text{A}}_{595}\right]}{{\text{A}}_{595\left(\text{blank}\right)}}\times 200\left[\text{μg}\right] \end{array}$$

#### Water retention ability of DMPs

The oxidized protein precipitate was weighed (recorded as m_1_) and transferred to an empty centrifuge tube (recorded as m_2_). Samples were heated at 80°C for 2 h, then cooled to atmospheric temperature, and weighed again (recorded as m_3_). The water retention ability of the oxidized protein precipitate sample was calculated according to Equation (4). The average value was obtained by repeating three times for each treated samples.


(4) $$\begin{array}{l} \text{Water retention ability}=\frac{{\text{m}}_{3}\text{-}{\text{m}}_{2}}{{\text{m}}_{1}}\times 100\text{\%} \end{array}$$

#### SDS-PAGE and myosin heavy chain immunoblot

Sample preparation and electrophoresis were conducted as described by Laemmli (17), with appropriate modifications. The oxidized protein precipitate obtained above was dissolved in phosphate buffer (0.6 mol/L NaCl containing 10 mmol/L K_2_HPO_4_, pH 7.0), and the concentration of the sample was adjusted to 2 mg/mL. The protein solution was mixed with sample buffer (NuPAGE™, Invitrogen, Carlsbad, CA, USA) as well as dithiothreitol (DTT) or without DTT and heated at 100°C for 2 min. After electrophoresis, the gel was stained with Coomassie brilliant blue G-250.

After electrophoresis, immunoblotting was performed using a Mini Cell SureLock equipped with an XCell II Blot Module (Invitrogen). The peptides were transferred to a cellulose acetate membrane, and the membrane was incubated with myosin heavy chain (MHC) rabbit polyclonal antibody (Beyotime, Shanghai, China). The membrane was washed and incubated with conjugated secondary antibody, alkaline phosphatase-labeled goat anti-rabbit IgG (H+L) (Beyotime), at 20°C for 1 h. Finally, a BCIP/NBT alkaline phosphatase color development kit (Beyotime) was used to develop the blots. Images were then photoed for analysis.

### Gelation and gel-forming ability parameter measurements

#### Preparation of duck myofibrillar proteins gel

A heat-induced gel formed from oxidized DMPs was prepared as follows. The oxidized protein precipitate was dissolved in phosphate buffer (0.6 mol/L NaCl containing 10 mmol/L K_2_HPO_4_, pH 7.0), adjusted to 30 mg/mL, heated at 85°C for 45 min, cooled in ice water, and then placed at 4°C overnight until use.

#### Gel strength measurements

The strength of the DMPs gel was determined based on the method reported by Zhu et al. (18) using a texture analyzer (TA-XT plus Plaser, Stable Micro Systems, Godalming, UK) with a p/5 probe. The samples were subjected to a double compression cycle at a probe depth of 5 mm, pre-test rate of 0.5 mm/s, test rate of 0.5 mm/s, and post-test rate of 0.5 mm/s. Three parallel tests were performed for each treatment.

#### Dynamic rheological analysis

Dynamic rheological tests of the oxidized DMPs solution were performed using a rheometer (MCR-301, Anton Paar, Graz, Austria) in oscillatory mode. The oxidized protein precipitate was dissolved in 0.6 mol/L NaCl containing 10 mmol/L K_2_HPO_4_ (pH 7.0) and adjusted to 30 mg/mL. The instrument was initialized prior to use. The rheological parameters were as follows: selected fixture: 50 mm plate, gap: 1 mm, scan range: 25–80°C, temperature increase rate: 2°C /min, hold at 80°C for 5 min, cool at 5°C /min, oscillation frequency: 0.1 Hz, strain: 2%. Changes in the storage modulus (G′) and loss modulus (G″) during temperature scanning were recorded.

#### Water holding capacity and low-field nuclear magnetic resonance measurements

The water-holding capacity (WHC) of the DMPs gels was determined using the centrifugation method described by Salvador et al. (19). The nuclear magnetic resonance (NMR) spin relaxation time (T_2_) was determined as reported by Gravelle et al. (20) using an NMR analyzer (NMI20-040H-I, Niumag Electric Co., Shanghai, China) with appropriate modifications. The conditions were set as follows: resonance frequency, 40 MHz; sampling number, 8; wait time, 2,000 ms; number of echoes, 9,000; scan range, 0–10,000 ms; each treatment group was measured three times in parallel. The data were inverted in batches using the data query option.

#### Protein secondary structure content analysis

The secondary structure of the DMPs gels was determined as described by Chen et al. (21) using a Labram HR800 spectrometer (Horiba Jobi Yvon S.A.S., Longjumeau, France) with a 532 nm argon ion laser and 600 mm grating. The conditions were as follows: scan range of the raman spectra: 400–3,600 cm^−1^ with a resolution of 1 cm^−1^, an acquisition time of 30 s, and a cumulative number of acquisitions: 2. The spectra were standardized with the phenylalanine band at 1,003 cm^−1^ using LabSpec version 5 (Horiba Jobi Yvon, Longjumeau, France). Secondary structure content was calculated as described by Alix et al. (22) according to changes in the amide I band with 3 repetitions for each treated group.

#### Microstructure analysis

The microstructure of the oxidized DMPs gels was determined using a Hitachi S-3000N scanning electron microscope (Tokyo, Japan) at an accelerating voltage of 20 kV. Briefly, the gels were cut to 0.5 × 0.5 × 0.2 cm in size, fixed in glutaraldehyde at a concentration of 4% for 24 h at 4°C, and washed three times with phosphate buffer for 10 min each time. The samples were dehydrated in a gradient of 50, 70, 80, and 90% ethanol for 15 min at each concentration and in 100% ethanol for 30 min. The samples were taped to the sample table, plated with a 10 nm gold film on the observation surface by ion sputtering, evaluated using scanning electron microscopy to determine the microstructure of the DMPs gels, and photographed.

### Statistical analysis

Data were processed using SPSS software (version 20.0, SPSS, Inc., Chicago, IL, USA) and subjected to one-way analysis of variance with Duncan's multiple range test for statistical analysis.

## Results and discussion

### Effects of oxidation on physicochemical changes

**Figure 1:**
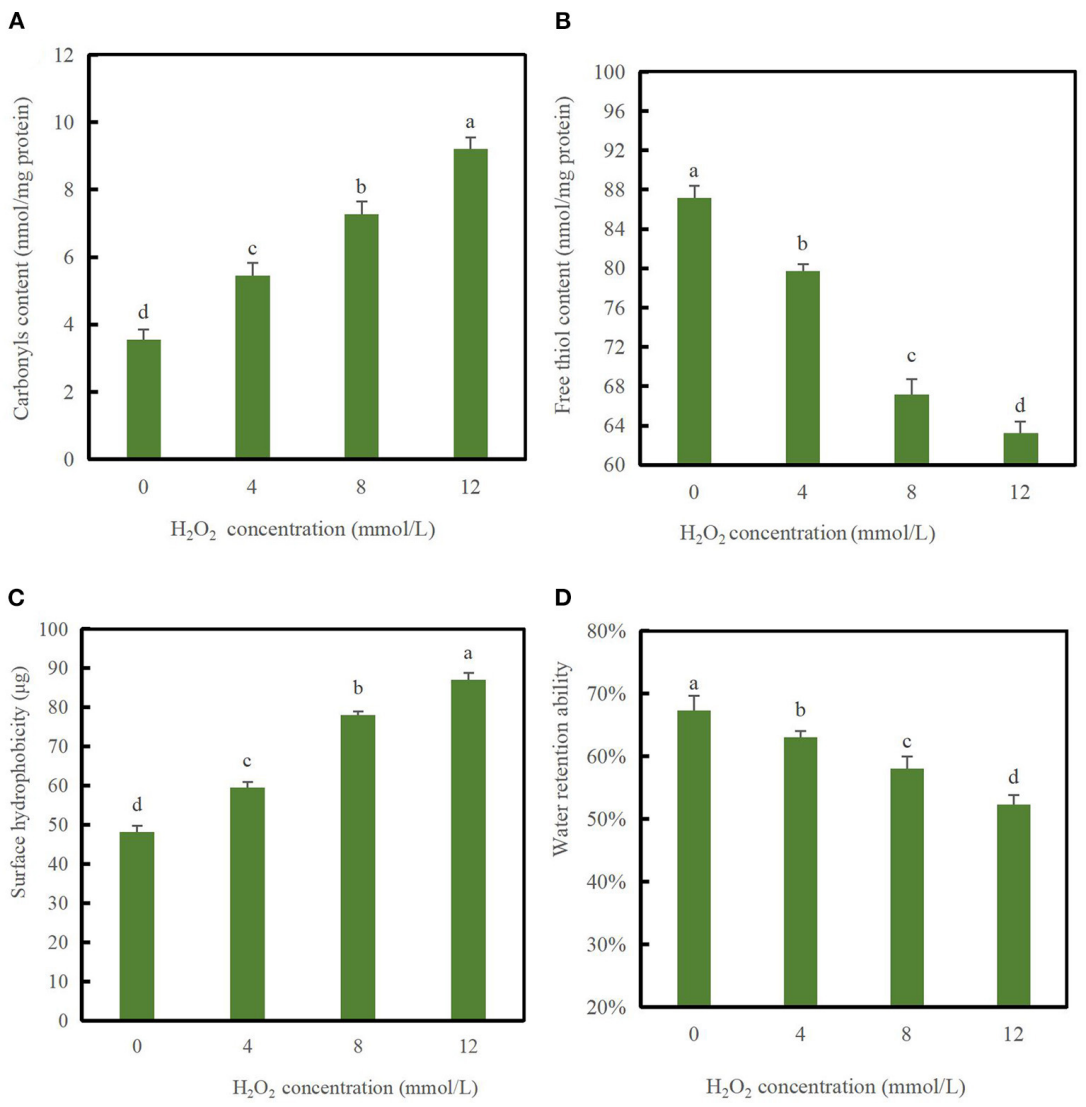
Changes in carbonyl values **(A)**, free thiol content **(B)**, surface hydrophobicity **(C)**, and water retention ability **(D)** of duck myofibrillar proteins (DMPs) treated with various hydroxyl radical levels (H_2_O_2_ concentrations: 0, 4, 8, 12 mmol/L). Different lowercase letters (a–d) indicate significant differences between the treated and control groups (*p* < 0.05).

The carbonyl content can be used as an indicator of protein oxidation (23). As shown in Figure 1A, following treatment with H_2_O_2_, the carbonyl content in DMPs varied from 5.45 to 9.21 nmol/mg protein. Furthermore, with increases in the hydroxyl radical strength, the DMPs carbonyl content increased significantly, indicating that hydroxyl radicals had dose-dependent effects on the carbonyl content from 0 to 12 mmol/L H_2_O_2_. The amino groups of DMPs with [[Inline Image]]NH[[Inline Image]], =NH, or [[Inline Image]]NH_2_ on the side chain are very sensitive to ·OH and were easily converted into carbonyl groups through oxidative deamination reactions (24, 25).

As shown in Figure 1B, the content of free thiol groups decreased significantly (*p* < 0.05) with increasing H_2_O_2_ concentrations and decreased to the minimum value of 63.24 nmol/mg protein when H_2_O_2_ reached 12 mmol/L compared to the blank group, indicating that protein oxidation had occurred. The SH content of the blank group was 87.16 nmol/mg protein, which was similar to that of porcine myofibrillar proteins (1). The reason for the significant decrease in the content of the free thiol group might be related to the fact that thiol residues in sulfhydryl-containing amino acids are extremely easily oxidized, and hydroxyl radicals react with free thiol groups and eventually generate disulfide bonds. Alternatively, protein sulfhydryl groups undergo complex changes following the oxidation of hydroxyl radicals to generate reversible or irreversible oxidation products (26). Myosin is rich in SH groups that can be readily converted to disulfide linkages upon oxidative stress (27). Therefore, myosin may form disulfide bonds following oxidation by hydroxyl radicals as a significant decrease in the content of free thiol groups was observed in our study (28).

As shown in Figure 1C, the surface hydrophobicity index was 48.19 μg in the absence of hydroxyl radicals. As H_2_O_2_ concentrations increased from 0 to 12 mmol/L, the surface hydrophobicity of the DMPs increased significantly (*p* < 0.05) and reached a maximum value of 87.13 μg when the H_2_O_2_ concentrations was 12 mmol/L. This result was significantly different from that in the blank group (*p* < 0.05), suggesting that oxidation at high concentrations induced further protein unfolding. These results agree with those reported previously, protein unfolding and loss of MHC of porcine myofibrillar proteins occurred continuously with increasing H_2_O_2_ concentrations (13). In summary, a higher content of oxidation radicals results in more denaturation of partial myofibrillar proteins, leading to more unfolded spatial conformational changes and exposure of a larger number of hydrophobic side chain amino acid residues to the polar water environment during oxidation (29). Oxidative damage may have induced partial unfolding of DMPs, thereby exposing hydrophobic amino acids that are normally buried inside protein molecules (30). This speculation agrees with the finding of Li et al. (31), who also observed that oxidation enhanced the surface hydrophobicity of porcine myofibrillar proteins.

Figure 1D showed that the water retention ability of the DMPs decreased with increasing H_2_O_2_ concentrations (*p* < 0.05). In the absence of hydroxyl radicals, the water retention ability of the DMPs was 67.33%, which decreased markedly to 52.33% following treatment with 12 mmol/L H_2_O_2_ (*p* < 0.05). Water loss was related to the influence of the intact myofibril structure. A possible explanation for the significant decrease in the water retention ability is that under attack by a high concentration of hydroxyl radicals, some protein aggregates with larger molecular weight among DMPs through disulfide bonds, leading to structural damage and a reduced ability to retain water molecules. This speculation is consistent with the electrophoretic results as described in section Myofibril protein profiles below. Protein oxidation in meat alters has been suggested to alter its electronic arrangement, thereby affecting the chemical interaction between myofibrillar proteins and water molecules (30). Generally, the effect of protein oxidation on cross-linking and aggregation is among the most important factors influencing the water retention ability (32).

### Myofibril protein profiles

**Figure 2:**
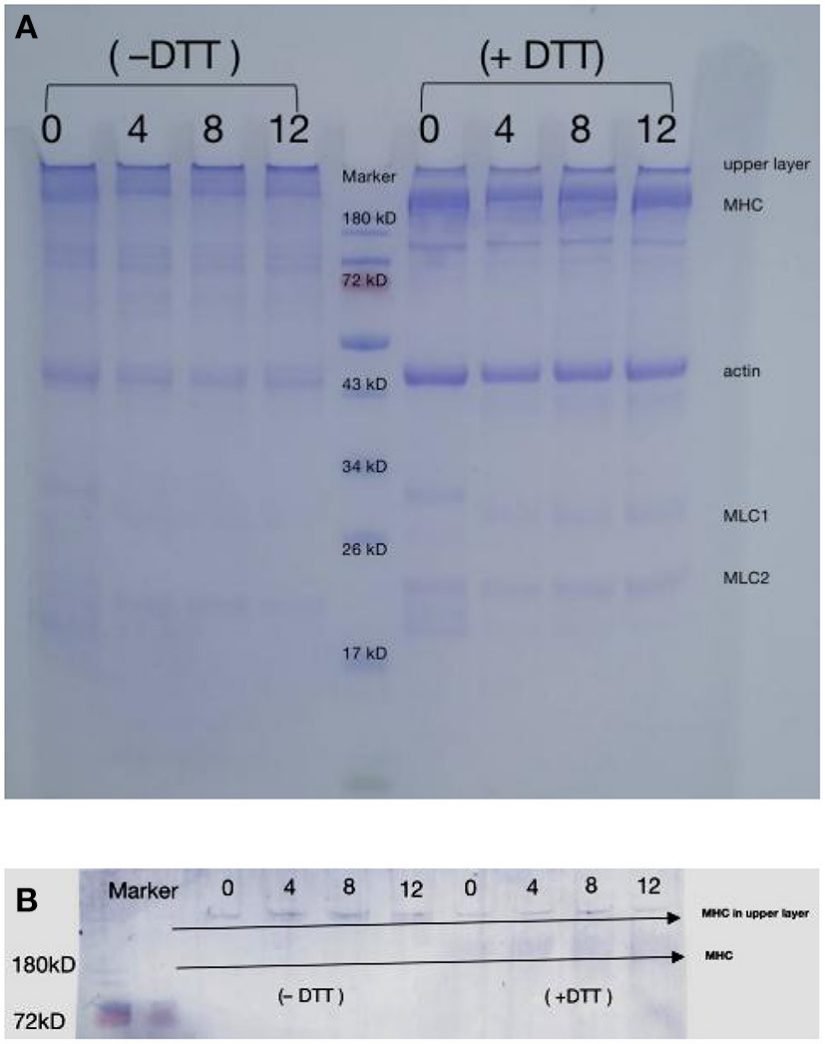
Changes in SDS-PAGE patterns **(A)** and myosin heavy chain immunoblot **(B)** of duck myofibrillar proteins treated with various hydroxyl radical levels (H_2_O_2_ concentrations: 0, 4, 8, 12 mmol/L). DTT means treated sample without dithiothreitol (DTT); + DTT means treated sample with dithiothreitol (DTT).

SDS-PAGE was performed to detect oxidation-induced covalent aggregation of the DMPs. The samples were prepared with or without DTT to determine the involvement of disulfide bonds in aggregation. As shown in Figure 2A, protein bands were observed at approximately 220, 45, and 22 kDa, corresponding to MHC, actin, and myosin light chain, respectively (13). The SDS-PAGE patterns did not reveal major differences in the protein composition between samples. However, the intensity of MHC at 220 kDa appeared to be much weaker in the 12 mmol/L-treated group than in the blank group, which might reflect a decrease in the solubility of myosin over time as a result of protein cross-linking, as previously suggested (33, 34).

In the absence of DTT, DMPs were oxidized, and the intensity of the myosin and actin bands decreased slightly, whereas more cross-linked polymers appeared in the upper layer of the sampling well. These results revealed that oxidation by high concentrations of hydroxyl radicals causes amino acid side-chain groups within the protein or those on different peptide chains to aggregate. The formation of protein aggregates increased the molecular weight of proteins, causing the protein to remain in the loading gel (15). The large reduction in protein aggregates at the top of the gel in the presence of DTT indicated that the aggregates were formed through disulfide bonds oxidized by SH with MHC, which is consistent with the western blotting results (Figure 2B) and agrees with those of a previous study showing that MHC is cross-linked through disulfide bonds during oxidation (35). Shen et al. (9) also reported that porcine MHC, α-actinin, and actin participate in aggregate formation after oxidation. However, myosin fragmentation was not observed under the present oxidative conditions (36), possibly because lower concentrations of H_2_O_2_ were used in the present study (0–12 mmol/L). Figures 2A,B clearly showed that after the addition of DTT, protein aggregates remained at the top of the gel, indicating that protein-protein cross-linking also occurred *via* other non-disulfide covalent bonds, such as Tyr-Tyr and carbonyl-NH_2_ interactions (37).

### Gel properties analysis

**Figure 3:**
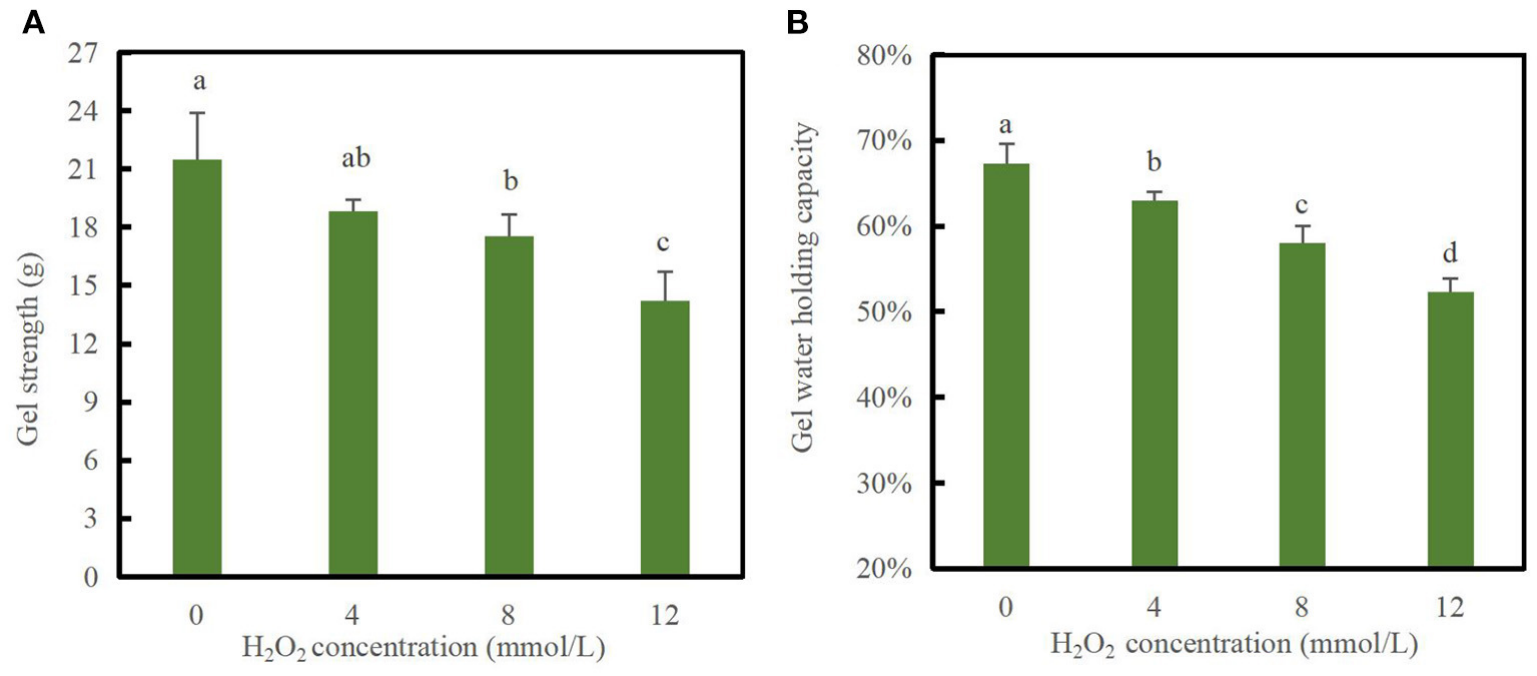
Changes in gel strength **(A)** and water-holding capacity **(B)** of duck myofibrillar protein (DMPs) gels pretreated with various hydroxyl radical levels (H_2_O_2_ concentrations: 0, 4, 8, 12 mmol/L). Different lowercase letters (a–d) indicate significant differences between the treatment and control groups (*p* < 0.05).

Gel strength is a good indicator of the structural integrity of proteins and their ability to form a gel mesh. The process of forming a gel mesh by thermal induction of DMPs involves denaturation of proteins by increased temperature, mutual agglutination between proteins, and mutual cross-linking, each of which affects the properties of the gel (12). As shown in Figure 3A, the gel strength decreased significantly (*p* < 0.05) with increasing concentrations of H_2_O_2_. For example, when the hydroxyl radical concentration reached 12 mmol/L, the gel strength decreased to a minimum value of 14.19 g, which was 33.9% lower than that of the blank group (*p* < 0.05). This result indicates that the textural properties of DMPs gels decreased after oxidation with hydroxyl radicals, and the significant decrease in gel strength was likely due to cross-linking of proteins caused by oxidation, which disrupted the conformation and was not conducive to the formation of an ordered gel mesh. This was further supported by the continuous loss of MHC in the aforementioned SDS-PAGE profile results, which is consistent with the gel microstructure results discussed below. Notably, a previous study demonstrated that the texture parameters of chicken DMPs gel from myofibrillar protein treated with 0–10 mmol/L H_2_O_2_ could not be measured; however, those of duck meat gel could be measured. This result may be related to differences in the animal species. Generally, excessive disulfide cross-links in oxidized myofibrillar proteins may hinder the ordered interactions of reactive functional groups and cause the reduction of hydrogen bonds in the protein matrix, which can eventually decrease the degree of association between proteins and proteins or water molecules, thus inhibiting the formation of a stable three-dimensional network structure (38). Notably, oxidation modification by hydroxyl radicals is also not favorable for gel whiteness. Compared with the blank group, the whiteness of the 12 mmol/L group decreased by 19.7% (data not shown), indicating the occurrence of structural changes that eventually led to decreased light scattering and thus a decreased gel whiteness value.

The WHC is a quantitative indicator of water retained within the structure of the protein gel network and can reflect the spatial structure of protein gels (39). As shown in Figure 3B, the gel WHC significantly decreased with increasing H_2_O_2_ concentrations (*p* < 0.05). At hydroxyl radical concentrations of 0, 4, 8, and 12 mmol/L, the gel WHC values were 67.33, 63.02, 58.04, and 52.33%, respectively. When the H_2_O_2_ concentration reached 12 mmol/L, the WHC of the gels decreased by 22.28% compared to that of the blank group. Oxidation modification caused by hydroxyl radicals induced DMPs cross-linking and made the proteins less likely to form a gel mesh structure, decreasing the capillary effect and making it difficult to retain water (1). Xiong et al. (40) reported that the WHC of myofibrillar protein gels was not affected by oxidation at relatively low H_2_O_2_ concentrations (≤0.5 mmol/L), whereas the WHC markedly decreased upon oxidation at high H_2_O_2_ concentrations. In addition, Li et al. (38) confirmed that the WHC of common carp myofibrillar protein gels was reduced after H_2_O_2_-mediated oxidation, which was consistent with the gel status and microstructure changes.

### Low-field nuclear magnetic resonance analysis

**Figure 4:**
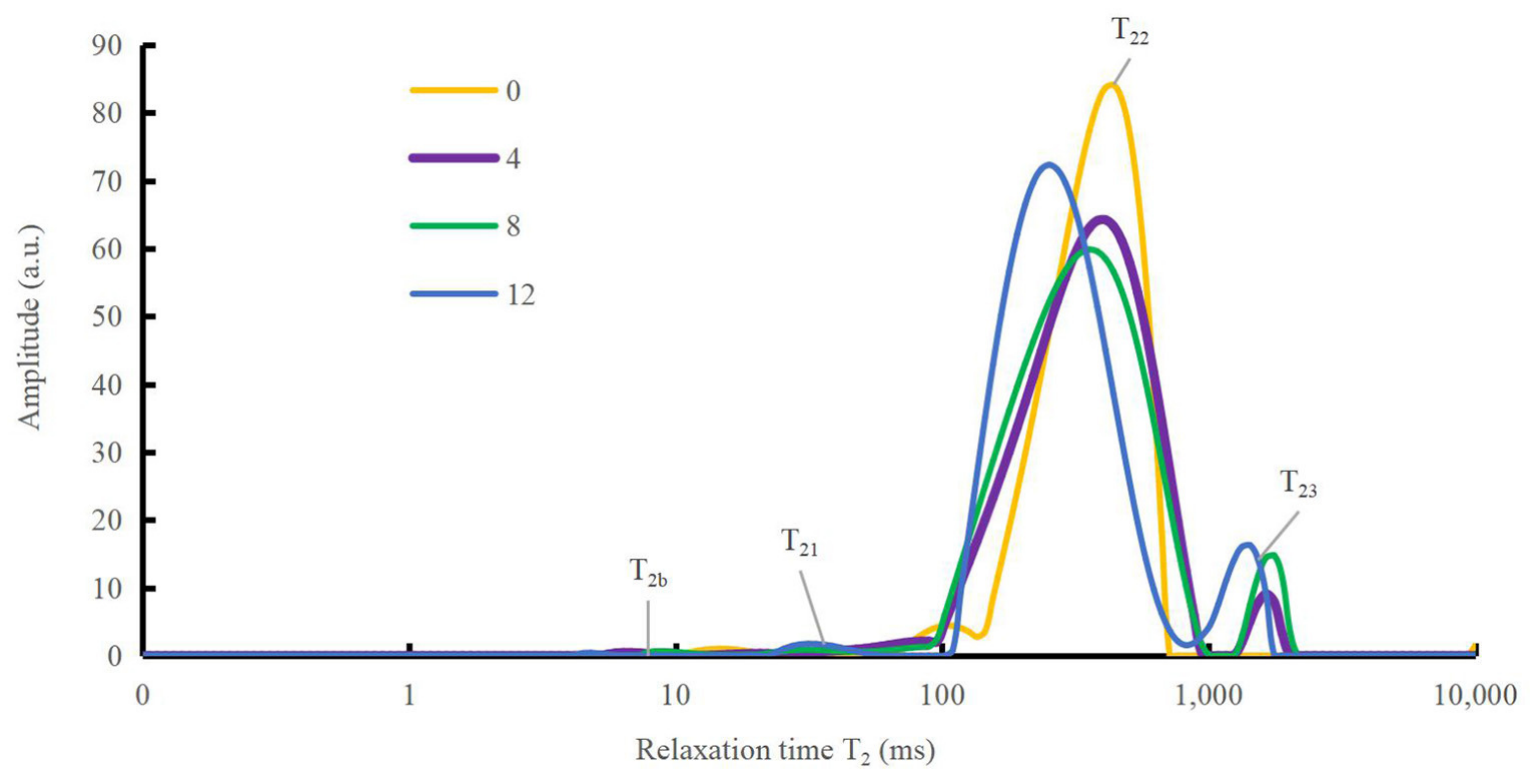
Variation in spin relaxation time (T_2_) of water molecules in duck myofibrillar proteins (DMPs) gels pretreated with various hydroxyl radical levels (H_2_O_2_ concentrations: 0, 4, 8, 12 mmol/L). T_2b_, T_21_, T_22_, and T_23_ denote bound, immobilized, moderately immobilized, and free water, respectively.

To better understand the mechanism of how oxidation affects water-holding properties, the water status of the DMPs was determined, as shown in Figure 4. The distribution and movement of water in the DMPs gel system can be expressed using the NMR proton spin relaxation time (T_2_). Han et al. (41) detected four peaks in the fitted NMR relaxation spectrum of myofibrillar protein gels, which correspond to four states of water (bound water, immobilized water, moderately immobilized water, and free water), and the second and third peaks are commonly treated as immobilized water (42). The peak relaxation times of the DMPs gels after adding hydroxyl radicals are listed in Table 1. A shorter peak relaxation time indicates that water is bound more tightly. T_22_ indicates immobilized water with a peak relaxation time between 100 and 500 ms. T_23_ indicates free water with a peak relaxation time after 500 ms. The effect of increasing the hydroxyl radical concentration did not significantly affect the peak relaxation times of T_2b_, T_21_, and T_22_. However, the effect on the peak relaxation time of T_23_ was significant (*p* < 0.05). As shown in Table 1, with increasing concentrations of hydroxyl radicals, the effect on the ratio of the peak areas of T_22_ and T_23_ was more obvious and significant (*p* < 0.05). The peak area ratio of T_22_ decreased to 90.51% and that of T_23_ increased to 7.78% when the concentration of hydroxyl radicals reached 12 mmol/L compared to those in the blank group. This result indicates that the amount of immobilized water in the gel decreased, and the amount of free water in the gel increased with increasing H_2_O_2_ concentrations. This result may have been obtained because the oxidation of DMPs by hydroxyl radicals is not conducive to the formation of a gel mesh structure (8), as the structure cannot lock the distribution of water during thermal induction, causing some immobilized water to dissociate from the substrate to free water (10). These results correspond to recent findings by Zhang et al. (43), who observed that as the degree of oxidation increased, more protein hydrophobic groups unfolded. This condition possibly avianized the binding ability of the proteins to water, in turn causing some of the immobilized water to transform into free water.

**Table 1:** Changes in peak relaxation times and the ratio of peak areas of water molecules in duck myofibrillar proteins (DMPs) gels pretreated with various hydroxyl radicals levels (H_2_O_2_ concentrations: 0, 4, 8, 12 mmol/L).

**H_2_O_2_ concentration/mmol/L**	**0**	**4**	**8**	**12**
T_2b_ /ms	15.33^a^ ± 1.00	6.39^b^ ± 0.03	8.74^b^ ± 0.39	6.35^b^ ± 2.16
T_21_ /ms	99.31^a^ ± 4.29	16.41^b^ ± 3.05	27.44^b^ ± 2.83	20.33^b^ ± 7.86
T_22_ /ms	423.65^a^ ± 22.78	380.00^a^ ± 42.80	391.25^a^ ± 48.32	263.08^b^ ± 15.17
T_23_/ms	9841.33^a^ ± 224.40	1611.50^bc^ ± 46.92	1813.81^b^ ± 71.98	1331.45^c^ ± 141.44
P_2b_ /%	0.47^a^ ± 0.22	0.31^a^ ± 0.16	0.44^a^ ± 0.18	0.38^a^ ± 0.37
P_21_ /%	2.04^a^ ± 1.11	1.07^a^ ± 1.26	1.37^a^ ± 0.91	1.34^a^ ± 0.29
P_22_ /%	97.31^a^ ± 1.25	95.70^ab^ ± 1.62	92.74^bc^ ± 1.46	90.51^c^ ± 0.87
P_23_ /%	0.18^d^ ± 0.08	2.92^c^ ± 0.21	5.43^b^ ± 0.37	7.78^a^ ± 0.20

Different lowercase letters (a–d) between the treatment and control groups indicate significant differences (p <0.05); T_2b_, T_21_, T_22_, and T_23_ indicate the capping relaxation times of water molecules in different states of the gel. P_2b_, P_21_, P_22_, and P_23_ indicate the proportions of peak areas of water molecules in different gels.

### Rheological property analysis

**Figure 5:**
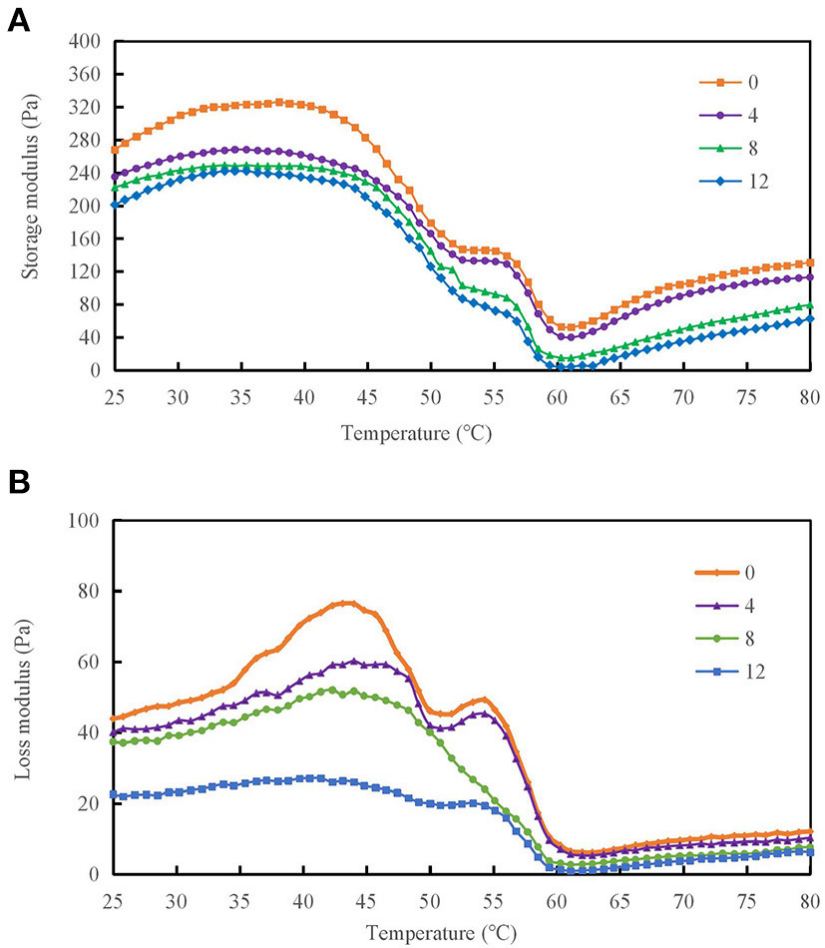
Variation in storage modulus **(A)** and loss modulus **(B)** of duck myofibrillar protein (DMPs) solution pretreated with various hydroxyl radical levels (H_2_O_2_ concentrations: 0, 4, 8, 12 mmol/L).

Rheological properties were measured for protein-protein interactions and the formation of gel mesh during the formation of gels from protein solutions (12). The storage modulus (G′) and loss modulus (G″) of the DMPs solution are important parameters of the rheological properties. Figure 5A shows that as the temperature increased, the G′ of the DMPs solution increased when different concentrations of H_2_O_2_ were added. When the temperature was increased to 38°C, the α-helix of the myosin head coalesced and initially formed a gel mesh structure, and G′ reached a maximum value. When the temperature was increased to 62°C, because of the breakage of myosin tail coalescence, the mobility of DMPs increased, and G′ decreased to the minimum value. When the temperature was further increased to 80°C, most of the myosin structures were stretched and coalescence occurred, leading to the formation of an ordered gel mesh structure, and G′ continued to slowly increase. The final G′ value decreased by 25.7% in the 12 mmol/L-treated group compared to the blank group. Therefore, oxidation is detrimental to gel formation in the reticular structure (37). Generally, G″ reflects viscous characteristics of DMPs. As the temperature was increased, the G″ of the DMPs solution also increased when different concentrations of hydroxyl radicals were added. When the temperature was increased to 43°C, G″ reached a maximum value because of the coalescence of the α-helix of the myosin head and the initial formation of the gel mesh structure. When the temperature was increased to 63°C, G″ decreased to the minimum value because of aggregation of the myosin tail. When the temperature was further increased to 80°C, G″ continued to increase slowly because of the stretching of most myosin structures and coalescence, leading to the formation of an orderly gel mesh structure (44). As shown in Figure 5B, the final G″ of the warming process significantly decreased by 65.7% when the hydroxyl radical concentration was 12 mmol/L compared to the value in the blank group (*p* < 0.05). As the concentration of hydroxyl radicals increased, myosin and actin in DMPs became severely cross-linked, which was not conducive to gel meshwork formation; therefore, G″ was also reduced.

### Analysis of the secondary structure

**Figure 6:**
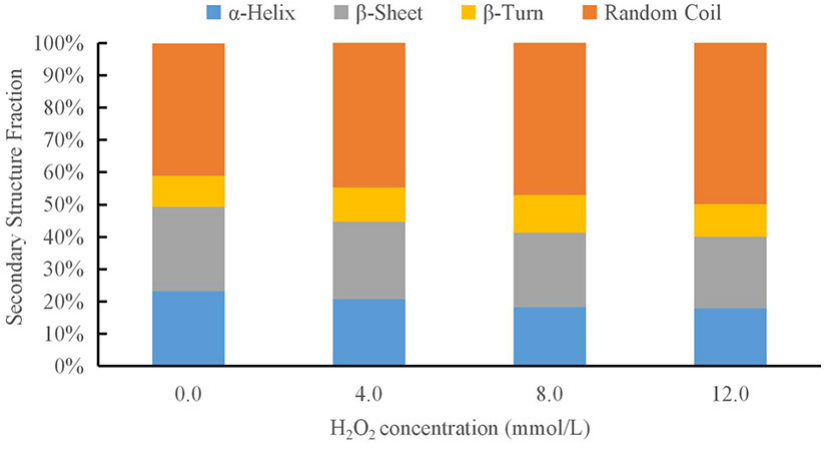
Changes in secondary structure composition of duck myofibrillar protein (DMPs) gels pretreated with various hydroxyl radicals levels (H_2_O_2_ concentrations: 0, 4, 8, 12 mmol/L).

The raman spectra of proteins include discrete bands that represent the vibrational modes of the peptide backbone and its side chains. The spectral position, intensity, and polarization of the raman bands are sensitive to protein secondary, tertiary, and quaternary structures, as well as side-chain orientation and the local environment (45). The secondary structure of the proteins was evaluated *via* Raman spectroscopy using the amide I (1,600–1,700 cm^−1^) band. Four different protein secondary structures were present in the control amide region, including α-helices, random coils, β-sheets, and β-turns, corresponding to the ranges 1,640–1,659, 1,660–1,669, 1,670–1,679, and 1,680–1,689 cm^−1^, respectively (44). The effect of different hydroxyl radical concentrations on the DMPs gel conformation was analyzed using raman spectroscopy, as shown in Figure 6. There were significant differences in the composition of the DMPs secondary structures treated with different concentrations of hydroxyl radicals. As the H_2_O_2_ concentration was increased, the percentage of α-helix area decreased, whereas the percentage of random coil area increased, and the β-sheet and β-turn remained relatively stable. The α-helix structure is mainly maintained by hydrogen bonds within the protein, and the hydroxyl radical attacks the amino group in the protein, which disrupts hydrogen bonds within the protein and eventually leads to a reduction in the α-helix structure, unfolding of the protein, and a gradual shift in protein structure from ordered to disordered (46). A previous study showed similar results, with an increase in myofibrillar protein aggregation. However, although the α-helix content decreased, the β-sheet content increased after oxidation (10). The decrease in α-helix content may, in turn, catalyze random coil transformation (21). Conversion of the secondary structure of DMPs caused by oxidation can lead to the unfolding of the myofibrillar protein structure, further enhancing the protein-protein interaction and thus affecting the gel properties (47).

### Microstructure analysis

**Figure 7:**
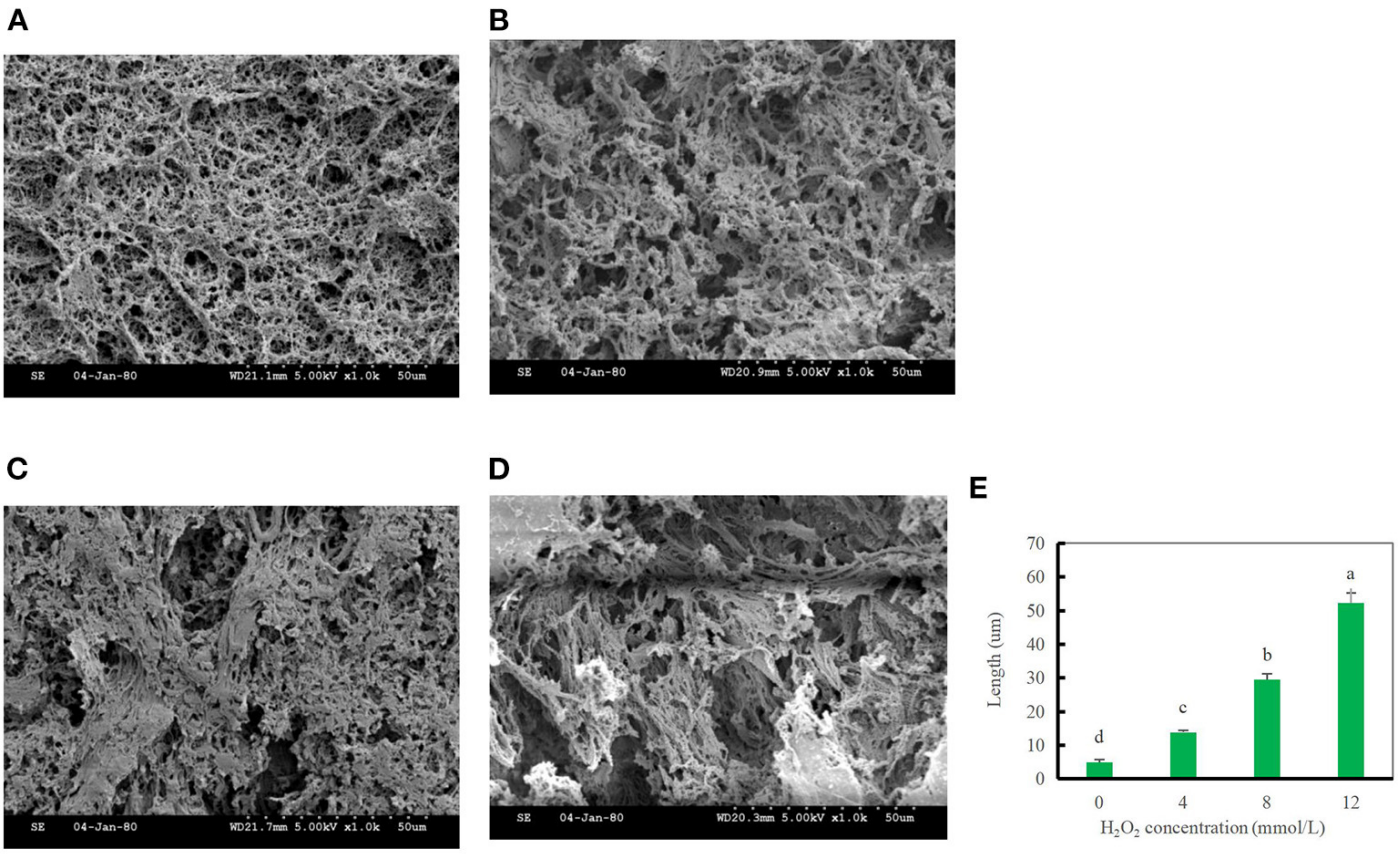
Changes in the gel microstructure of duck myofibrillar protein (DMPs) gels pretreated with 0 mmol/L **(A)**, 4 mmol/L **(B)**, 8 mmol/L **(C)**, 12 mmol/L H_2_O_2_ **(D)**, and changes in the pore diameter of DMPs gels **(E)** pretreated with various hydroxyl radicals levels.

The gel microstructure reflects the relationship between the gel properties and structure. In the blank group, myosin and actin rapidly stretched and coalesced to form a more uniform gel mesh structure. With increasing hydroxyl radical concentrations, the number of cavities in the gel decreased, and the mesh structure gradually changed from a uniform and tightly ordered to randomly distributed, loose, and porous. This result indicates that after oxidation modification by hydroxyl radicals, DMPs are not conducive to the formation of the gel mesh structure, as shown in Figure 7, which also explains the decrease in the WHC after oxidation at high concentrations of hydroxyl radicals (46). The diameter of the gel pores was significantly lower in the blank group than in the treated group, particularly when the hydroxyl radical concentration reached 12 mmol/L (Figure 7E). Oxidation of hydroxyl radicals alters the gel microstructure, possibly because hydroxyl radicals induce myosin and actin to cross-link in advance, as most of the myosin and actin are involved in forming the gel meshwork during thermally induced gel formation. It is also possible that the oxidation of high concentrations of hydroxyl radicals induced severe denaturation of the proteins, which disrupted the spatial conformation of the proteins and thus was not conducive to the formation of a gel meshwork, resulting in a disorganized, loose, and porous gel structure (13). This result was consistent with our previous finding of the gel strength.

## Conclusion

Oxidation by high concentrations of hydroxyl radicals causes changes in the physicochemical structure of DMPs. In addition, a greater degree of oxidation leads to more obvious changes in structural indices, combined with more cross-linking of proteins and the production of protein aggregation with higher molecular weights. Importantly, protein oxidation caused by hydroxyl radicals may not improve the gel properties, WHC, water status and distribution, or gel-forming capacity of DMPs. Therefore, the oxidation level of meat gel products should be carefully controlled during the handling, processing, and transportation of duck gel products. Further studies are needed to explore whether the stability of the gel properties of duck meat products can be improved using appropriate antioxidants. Our results provide a foundation for further studies of protein oxidation behavior in the processing of duck and duck meat products and its effects on meat products and industrial use.

## Data availability statement

The raw data supporting the conclusions of this article will be made available by the authors, without undue reservation.

## Author contributions

XZ: conceptualization, project administration, writing-review and editing, and methodology. XS: sample collection, investigation, data curation, and writing-original draft. SL: funding acquisition and visualization. YG and JL: funding acquisition and validation. QF and RW: supervision. All authors have read and agreed to the published version of the manuscript. All authors contributed to the article and approved the submitted version.

## Funding

This research was supported by the National Natural Science Foundation of China (31301507) and the Natural Science Fund for Colleges and Universities in Jiangsu Province (13KJB550006, 19KJB550005, and 21KJA550002).

## Conflict of interest

The authors declare that the research was conducted in the absence of any commercial or financial relationships that could be construed as a potential conflict of interest.

## Publisher's note

All claims expressed in this article are solely those of the authors and do not necessarily represent those of their affiliated organizations, or those of the publisher, the editors and the reviewers. Any product that may be evaluated in this article, or claim that may be made by its manufacturer, is not guaranteed or endorsed by the publisher.

## References

[1] Li Y Li X Wang JZ Zhang CH Sun HM Wang CQ. Effects of oxidation on water distribution and physicochemical properties of porcine myofibrillar protein gel. Food Biophys. (2014) 9:169–78. 10.1007/s11483-013-9329-9

[2] Wang H Luo Y Ertbjerg P. Myofibrillar protein gel properties are influenced by oxygen concentration in modified atmosphere packaged minced beef. Food Chem. (2017) 230:475–81. 10.1016/j.foodchem.2017.03.073 28407937

[3] Hellwig M. The chemistry of protein oxidation in food. Angew Chem Int Ed. (2019) 58:16742–63. 10.1002/anie.201814144 30919556

[4] Fu QQ Liu R Wang HO Hua C Song SX Zhou GH. Effects of oxidation *in vitro* on structures and functions of myofibrillar protein from beef muscles. J Agric Food Chem. (2019) 67:5866–73. 10.1021/acs.jafc.9b01239 31026156

[5] Deng X Lei Y Liu J Zhang J Qin J. Biochemical changes of Coregonus peled myofibrillar proteins isolates as affected by HRGS oxidation system. J Food Biochem. (2018) 43:e12710. 10.1111/jfbc.12710 31353664

[6] Liu C Li W Zhou M Yi S Ye B Mi H. Effect of oxidation modification induced by peroxyl radicals on the physicochemical and gel characteristics of grass carp myofibrillar protein. J Food Meas Charact. (2021) 15:5572–83. 10.1007/s11694-021-01123-1

[7] Bao Y Puolanne E Ertbjerg P. Effect of oxygen concentration in modified atmosphere packaging on color and texture of beef patties cooked to different temperatures. Meat Sci. (2016) 121:189–95. 10.1016/j.meatsci.2016.06.014 27341620

[8] Nyaisaba BM Liu X Zhu S Fan X Sun L Hatab S. Effect of hydroxyl-radical on the biochemical properties and structure of myofibrillar protein from Alaska pollock (Theragra chalcogramma). LWT. (2019) 106:15–21. 10.1016/j.lwt.2019.02.045

[9] Shen H Elmore JS Zhao M Sun W. Effect of oxidation on the gel properties of porcine myofibrillar proteins and their binding abilities with selected flavour compounds. Food Chem. (2020) 329:127032. 10.1016/j.foodchem.2020.127032 32505986

[10] Li DY Tan ZF Liu ZQ Wu C Liu HL Guo C. Effect of hydroxyl radical induced oxidation on the physicochemical and gelling properties of shrimp myofibrillar protein and its mechanism. Food Chem. (2021) 351:129344. 10.1016/j.foodchem.2021.129344 33647688

[11] Wang J Yang Y Tang X Ni W Zhou L. Effects of pulsed ultrasound on rheological and structural properties of chicken myofibrillar protein. Ultrason Sonochem. (2017) 38:225–33. 10.1016/j.ultsonch.2017.03.018 28633822

[12] Han Y Shen H Zhao M Sun W. Changes in structural and gel properties of myofibrillar proteins induced by sodium chloride and hydroxyl radical. Food Sci Technol Res. (2019) 25:97–106. 10.3136/fstr.25.97

[13] Park D Xiong YLL Alderton AL. Concentration effects of hydroxyl radical oxidizing systems on biochemical properties of porcine muscle myofibrillar protein. Food Chem. (2007) 101:1239–46. 10.1016/j.foodchem.2006.03.028

[14] Soglia F Petracci M Ertbjerg P. Novel DNPH-based method for determination of protein carbonylation in muscle and meat. Food Chem. (2016) 197:670–5. 10.1016/j.foodchem.2015.11.038 26617002

[15] Bao Y Boeren S Ertbjerg P. Myofibrillar protein oxidation affects filament charges, aggregation and water-holding. Meat Sci. (2018) 135:102–8. 10.1016/j.meatsci.2017.09.011 28968552

[16] Chelh I Gatellier P Santé-Lhoutellier V. Technical note: a simplified procedure for myofibril hydrophobicity determination. Meat Sci. (2006) 74:681–3. 10.1016/j.meatsci.2006.05.019 22063223

[17] Laemmli UK. Cleavage of structural proteins during the assembly of the head of bacteriophage T4. Nature. (1970) 227:680–5. 10.1038/227680a0 5432063

[18] Zhu X Tan B Li K Liu S Gu Y Xia T. The impacts of different pea protein isolate levels on functional, instrumental and textural quality parameters of duck meat batters. Foods. (2022) 11:1620. 10.3390/foods11111620 35681371 PMC9180532

[19] Salvador P Toldra M Saguer E Carretero C Pares D. Microstructure-function relationships of heat-induced gels of porcine haemoglobin. Food Hydrocoll. (2009) 23:1654–9. 10.1016/j.foodhyd.2008.12.003

[20] Gravelle AJ Marangoni AG Barbut S. Insight into the mechanism of myofibrillar protein gel stability: influencing texture and microstructure using a model hydrophilic filler. Food Hydrocoll. (2016) 60:415–24. 10.1016/j.foodhyd.2016.04.014

[21] Chen J Deng T Wang C Mi H Yi S Li X. Effect of hydrocolloids on gel properties and protein secondary structure of silver carp surimi. J Sci Food Agric. (2020) 100:2252–60. 10.1002/jsfa.10254 31917477

[22] Alix AJP Pedanou G Berjot M. Fast determination of the quantitative secondary structure of proteins by using some parameters of the Raman Amide I band. J Mol Struct. (1988) 174:159–64. 10.1016/0022-2860(88)80151-0

[23] Estevez M Ventanas S Cava R. Protein oxidation in frankfurters with increasing levels of added rosemary essential oil: effect on color and texture deterioration. J Food Sci. (2005) 70:C427–32. 10.1111/j.1365-2621.2005.tb11464.x

[24] Stadtman ER Levine RL. Free radical-mediated oxidation of free amino acids and amino acid residues in proteins. Amino Acids. (2003) 25:207–18. 10.1007/s00726-003-0011-2 14661084

[25] Eymard S Jacobsen C Baron CP. Assessment of washing with antioxidant on the oxidative stability of fatty fish mince during processing and storage. J Agric Food Chem. (2010) 58:6182–9. 10.1021/jf904013k 20423096

[26] Sante-Lhoijtellier V Astrijc T Marinova P Greve E Gatellier P. Effect of meat cooking on physicochemical state and *in vitro* digestibility of myofibrillar proteins. J Agric Food Chem. (2008) 56:1488–94. 10.1021/jf072999g 18237130

[27] Cao Y True AD Chen J Xiong YL. Dual role (anti- and pro-oxidant) of gallic acid in mediating myofibrillar protein gelation and gel *in vitro* digestion. J Agric Food Chem. (2016) 64:3054–61. 10.1021/acs.jafc.6b00314 27003685

[28] Liu G Xiong YLL. Electrophoretic pattern, thermal denaturation, and *in vitro* digestibility of oxidized myosin. J Agric Food Chem. (2000) 48:624–30. 10.1021/jf990520h 10725125

[29] Riebroy S Benjakul S Visessanguan W Erikson U Rustad T. Acid-induced gelation of natural actomyosin from Atlantic cod (Gadus morhua) and burbot (Lota lota). Food Hydrocoll. (2009) 23:26–39. 10.1016/j.foodhyd.2007.11.010 26054263

[30] Estevez M. Protein carbonyls in meat systems: a review. Meat Sci. (2011) 89:259–79. 10.1016/j.meatsci.2011.04.025 21621336

[31] Li C Xiong YL Chen J. Oxidation-induced unfolding facilitates myosin cross-linking in myofibrillar protein by microbial transglutaminase. J Agric Food Chem. (2012) 60:8020–7. 10.1021/jf302150h 22809283

[32] Bao Y Ertbjerg P. Relationship between oxygen concentration, shear force and protein oxidation in modified atmosphere packaged pork. Meat Sci. (2015) 110:174–9. 10.1016/j.meatsci.2015.07.022 26241463

[33] Eymard S Baron CP Jacobsen C. Oxidation of lipid and protein in horse mackerel (Trachurus trachurus) mince and washed minces during processing and storage. Food Chem. (2009) 114:57–65. 10.1016/j.foodchem.2008.09.03020423096

[34] Baron CP Kjaersgard IVH Jessen F Jacobsen C. Protein and lipid oxidation during frozen storage of rainbow trout (Oncorhynchus mykiss). J Agric Food Chem. (2007) 55:8118–25. 10.1021/jf070686f 17713921

[35] Wang BW Xiong YLL. Functional stability of antioxidant-washed, cryoprotectant-treated beef heart surimi during frozen storage. J Food Sci. (1998) 63:293–8. 10.1111/j.1365-2621.1998.tb15729.x

[36] Ooizumi T Xiong YL. Biochemical susceptibility of myosin in chicken myofibrils subjected to hydroxyl radical oxidizing systems. J Agric Food Chem. (2004) 52:4303–7. 10.1021/jf035521v 15212484

[37] Cao Y Ma W Wang J Zhang S Wang Z Zhao J. Influence of sodium pyrophosphate on the physicochemical and gelling properties of myofibrillar proteins under hydroxyl radical-induced oxidative stress. Food Funct. (2020) 11:1996–2004. 10.1039/C9FO02412C 32101205

[38] Li Y Kong B Xia X Liu Q Diao X. Structural changes of the myofibrillar proteins in common carp (*Cyprinus carpio*) muscle exposed to a hydroxyl radical-generating system. Process Biochem. (2013) 48:863–70. 10.1016/j.procbio.2013.03.015

[39] Lu H Zhang L Li Q Luo Y. Comparison of gel properties and biochemical characteristics of myofibrillar protein from bighead carp (Aristichthys nobilis) affected by frozen storage and a hydroxyl radical-generation oxidizing system. Food Chem. (2017) 223:96–103. 10.1016/j.foodchem.2016.11.143 28069130

[40] Xiong YL Blanchard SP Ooizumi T Ma Y. Hydroxyl radical and ferryl-generating systems promote gel network formation of myofibrillar protein. J Food Sci. (2010) 75:C215–21. 10.1111/j.1750-3841.2009.01511.x 20492228

[41] Han M Wang P Xu X Zhou G. Low-field NMR study of heat-induced gelation of pork myofibrillar proteins and its relationship with microstructural characteristics. Int Food Res J. (2014) 62:1175–82. 10.1016/j.foodres.2014.05.062

[42] Qin H Xu P Zhou C Wang Y. Effects of l-Arginine on water holding capacity and texture of heat-induced gel of salt-soluble proteins from breast muscle. LWT. (2015) 63:912–8. 10.1016/j.lwt.2015.04.048

[43] Zhang D Li H Emara AM Hu Y Wang Z Wang M. Effect of *in vitro* oxidation on the water retention mechanism of myofibrillar proteins gel from pork muscles. Food Chem. (2020) 315. 10.1016/j.foodchem.2020.126226 32018081

[44] Zhu X Zhang J S Gu Y Yu X Gao F. Relationship between molecular structure and heat-induced gel properties of duck myofibrillar proteins affected by the addition of pea protein isolate. Foods. (2022) 11:1040. 10.3390/foods11071040 35407127 PMC8997435

[45] Benevides JM Overman SA Thomas GJ Jr. Raman spectroscopy of proteins. Curr Protoc Protein Sci. (2003) 33:17.8.1–35. 10.1002/0471140864.ps1708s33 18429253

[46] Ullah N Wang X Chen L Xu X Li Z Feng X. Influence of biofilm surface layer protein A (BslA) on the gel structure of myofibril protein from chicken breast. J Sci Food Agric. (2017) 97:4712–20. 10.1002/jsfa.8339 28374425

[47] Wang L Zhang M Fang Z Bhandari B. Gelation properties of myofibrillar protein under malondialdehyde-induced oxidative stress. J Sci Food Agric. (2017) 97:50–57. 10.1002/jsfa.7680 26916602

